# Genomic Characterization of Fluoroquinolone-Resistant Thermophilic *Campylobacter* Strains Isolated from Layer Chicken Feces in Gangneung, South Korea by Whole-Genome Sequencing

**DOI:** 10.3390/genes12081131

**Published:** 2021-07-25

**Authors:** Noel Gahamanyi, Dae-Geun Song, Kye-Yoon Yoon, Leonard E. G. Mboera, Mecky I. Matee, Dieudonné Mutangana, Erick V. G. Komba, Cheol-Ho Pan, Raghavendra G. Amachawadi

**Affiliations:** 1 Natural Product Informatics Research Center, KIST Gangneung Institute of Natural Products, Gangneung 25451, Korea; noel.gahamanyi@kist.re.kr (N.G.); dsong82@kist.re.kr (D.-G.S.); beautyygy@gmail.com (K.-Y.Y.); panc@kist.re.kr (C.-H.P.); 2 SACIDS Foundation for One Health, College of Veterinary Medicine and Biomedical Sciences, Sokoine University of Agriculture, Chuo Kikuu, Morogoro P.O. Box 3015, Tanzania; leonard.mboera@sacids.org (L.E.G.M.); ekomba@sua.ac.tz (E.V.G.K.); 3 Department of Veterinary Microbiology, Parasitology and Biotechnology, College of Veterinary Medicine and Biomedical Sciences, Sokoine University of Agriculture, Morogoro P.O. Box 3019, Tanzania; 4 School of Medicine, Muhimbili University of Health and Allied Sciences, Dar es Salaam P.O. Box 65001, Tanzania; mecky.matee@sacids.org; 5 College of Science and Technology, University of Rwanda, Kigali P.O. Box 3900, Rwanda; d.mutangana@ur.ac.rw; 6 Division of Bio-Medical Science and Technology, KIST School, Korea University of Science and Technology, Seoul 02792, Korea; 7 Department of Clinical Sciences, College of Veterinary Medicine, Kansas State University, Manhattan, KS 66506-5606, USA

**Keywords:** *Campylobacter*, layer chicken, fluoroquinolone-resistant, phylogenetic analysis, whole-genome sequencing, Korea

## Abstract

Thermophilic *Campylobacter* species of poultry origin have been associated with up to 80% of human campylobacteriosis cases. Layer chickens have received less attention as possible reservoirs of *Campylobacter* species. Initially, the minimum inhibitory concentration (MIC) and minimum bactericidal concentration (MBC) of two archived *Campylobacter* isolates (*Campylobacter jejuni* strain 200605 and *Campylobacter coli* strain 200606) from layer chickens to five antimicrobials (ciprofloxacin, nalidixic acid, erythromycin, tetracycline, and gentamicin) were determined using broth microdilution while the presence of selected antimicrobial resistance genes was performed by polymerase chain reaction (PCR) using specific primers. Whole-genome sequencing (WGS) was performed by the Illumina HiSeq X platform. The analysis involved antimicrobial resistance genes, virulome, multilocus sequence typing (MLST), and phylogeny. Both isolates were phenotypically resistant to ciprofloxacin (MIC: 32 vs. 32 µg/mL), nalidixic acid (MIC: 128 vs. 64 µg/mL), and tetracycline (MIC: 64 vs. 64 µg/mL), but sensitive to erythromycin (MIC: 1 vs. 2 µg/mL) and gentamicin (MIC: 0.25 vs. 1 µg/mL) for *C. jejuni* strain 200605 and *C. coli* strain 200606, respectively. WGS confirmed C257T mutation in the *gyr*A gene and the presence of *cme*ABC complex conferring resistance to FQs in both strains. Both strains also exhibited *tet*(O) genes associated with tetracycline resistance. Various virulence genes associated with motility, chemotaxis, and capsule formation were found in both isolates. However, the analysis of virulence genes showed that *C. jejuni* strain 200605 is more virulent than *C. coli* strain 200606. The MLST showed that *C. jejuni* strain 200605 belongs to sequence type ST-5229 while *C. coli* strain 200606 belongs to ST-5935, and both STs are less common. The phylogenetic analysis clustered *C. jejuni* strain 200605 along with other strains reported in Korea (CP028933 from chicken and CP014344 from human) while *C. coli* strain 200606 formed a separate cluster with *C. coli* (CP007181) from turkey. The WGS confirmed FQ-resistance in both strains and showed potential virulence of both strains. Further studies are recommended to understand the reasons behind the regional distribution (Korea, China, and Vietnam) of such rare STs.

## 1. Introduction

Worldwide, *C. jejuni* and *C. coli* are considered the leading etiologies of human campylobacteriosis [1,2]. Currently, most of the studies have focused on *C. jejuni,* which is associated with 85% of human infections [1]. However, *C. coli* has not received the same attention, but it is second to *C. jejuni* in causing human campylobacteriosis [2,3]. The major reservoirs include chickens and cattle, but other farm animals or food products and wild birds have been implicated in disease transmission [4,5,6]. Chicken ceca are colonized by high levels of *Campylobacter* which may persist in feces that are used as biofertilizers [7]. Human campylobacteriosis is of public health concern due to the increased number of *Campylobacter* strains that are resistant to both drugs of choice (macrolides and fluoroquinolones) and alternative therapies (aminoglycosides and tetracyclines) [8]. The missense mutation (C257T) in the quinolone resistance-determining region (QRDR) of *gyr*A has been associated with high-level resistance to quinolones [9]. The widespread FQ-resistant *C. jejuni* lineages via food and travel need urgent monitoring and mitigation strategies [10].

To control *Campylobacter*-related infections, it is necessary to understand virulence factors and molecular mechanisms contributing to pathogenesis [11,12]. WGS data from different pathogenic and non-pathogenic mutant strains have been used to classify virulence gene clusters linked to pathogenicity [13]. Although there are gaps in understanding the pathogenesis of *Campylobacter* [14], the roles played by several virulence factors involved in adhesion, invasion, chemotaxis, and motility are known [12,15]. However, there are various genes coding for other virulence factors, like the lipopolysaccharide (LPS), lipooligosaccharide (LOS), and capsule, which need to be well elucidated [12]. Several studies have confirmed the roles of some of the virulence genes by observing the limited capacities of mutants to attach to, colonize, and invade eukaryotic cells [15,16]. Mutant strains lacking *fla*A and *fla*B were unable to complete the colonization process in chickens [13,17]. Also, *cad*F and *cia*B mutant strains showed a reduced ability to adhere to and invade cell lines [17].

Multilocus sequence typing (MLST) has been the gold standard method used for epidemiological surveillance and source-attribution studies [18,19]. However, MLST does not include clinically important information, like the virulence or antibiotic resistance determinants, mobile genetic elements, nucleotide polymorphism, and other recombination events [20]. *Campylobacter* species can be well characterized based on their virulomes often acquired via horizontal gene transfer [21]. For instance, there are *C. coli* hybrid strains with DNA segments from *C. jejuni,* and MLST failed to genotype such strains [22].

Currently, WGS is considered the most informative and discriminative typing method of bacterial pathogens [2,23]. For instance, the WGS led to the creation of the core genome (cgMLST), a novel typing method encompassing hundreds of loci from the traditional seven loci [24]. Additionally, studies using single nucleotide polymorphism (SNP) allow the establishment of the best phylogenetic relationship among different pathogens [25]. The WGS is used for various purposes including novel antibiotic and diagnostic test development, studying the emergence of antibiotic resistance, disease surveillance, and direct infection control measures in both clinical settings and communities [26]. Next-generation sequencing (NGS) technologies are preferred in pathogen typing due to affordable cost and reduced turnaround time [27]. The NGS systems available include Illumina Genome Analyzer (HiSeq, MiSeq), Life Technologies Ion Torrent, and the PacBio RX system [28]. However, the use of WGS daily in genotyping and pathogen characterization faces hurdles related to bioinformatics, like resources, lack of validated workflows, and expertise, which are all required for data analysis [25]. This makes the efficient use of WGS data in public health investigations very hard [29]. It is important to note that some countries like the US have incorporated the WGS in routine checking of human pathogens from clinical samples and food.

Despite the progress in understanding the complicated and multifactorial pathogenesis of *Campylobacter* as an enteric pathogen, there is a gap regarding the combination of phenotypic and genotypic characteristics [30]. Furthermore, several epidemiological studies have been carried out on *Campylobacter* species from broiler chickens [31], but there is a dearth of information on *Campylobacter* from layer chickens [7]. Layer chickens have been reported to be the source of antimicrobial-resistant *Campylobacter* strains [7,32]. The WGS allows for comprehensive phylogenetic analyses of several factors associated with virulence or antibiotic resistance [20]. Based on findings of the partial characterization of layer chicken-derived *Campylobacter* isolates, we hypothesize that the WGS-characterized isolates harbor various antimicrobial and virulence-related genes contributing to their pathogenicity. To the best of our knowledge, there are no previous reports of WGS data of *Campylobacter* from layers in South Korea. Hence, the objectives of this study were to genomically characterize two FQ-resistant *C. jejuni* and *C. coli* of layer chicken origin by WGS and to establish phylogenetic relationships of the two isolates to the existing ones.

## 2. Materials and Methods

### 2.1. Campylobacter Strains and Culture Conditions

The two *Campylobacter* strains used in this study were selected from our previously published research work [8]. For this experiment, preserved strains were revived by inoculating them onto Mueller Hinton Agar as previously described [33]. Subculturing was performed to get colonies free from glycerol.

### 2.2. Antimicrobial Susceptibility Testing

Antimicrobial susceptibility testing (AST) against five antimicrobials, including FQs, namely ciprofloxacin (CIP) and nalidixic acid (NAL) (0.25–512 µg/mL), macrolide (erythromycin or ERY) (0.06–64 µg/mL), aminoglycoside (gentamicin or GEN) (0.06–64 µg/mL) and tetracycline (TET) (0.125–1024 µg/mL) was performed by two-fold broth microdilution [34]. The optical density was recorded spectrophotometrically at 600 nm (Synergy HT; BioTek Instruments Inc., Winooski, VT, USA). The same protocol used in our previous study was followed for minimum inhibitory concentration (MIC) and minimal bactericidal concentration (MBC) determination [8]. The AST procedure was done in six replicates for reproducibility. The MIC was measured spectrophotometrically with a microplate reader (Synergy HT; BioTek Instruments Inc., Winooski, VT, USA) and confirmed by the addition of iodonitrotetrazolium chloride.

### 2.3. DNA Extraction, Species Confirmation, and Antimicrobial Resistance (AMR) Genes Detection

The genomic DNA was extracted from pure colonies using the Qiagen QIAamp^®^ PowerFecal^®^ Kit (Qiagen, Hilden, Germany) as per the manufacturer’s instructions. For genes specific for *Campylobacter* genus and species or genes associated with antimicrobial resistance [*tet*(O), *gyr*A, and *cme*B], PCR was performed using specific primers (Table 1). After electrophoresis, bands of PCR products were observed on a Dual UV Transilluminator (Core Bio System, Huntington Beach, CA, USA) under ultraviolet (UV) light. Bands were compared to the 100 bp marker (Dyne bio, Seongnam-si, Korea). PCR products were purified with AMPure XP beads (Beckman Coulter, Fullerton, CA, USA) and sequenced by the Sanger method at SolGent (Solutions for Genetic Technologies, Daejeon, South Korea). The presence of resistance genes, as well as point mutations in the 23S rRNA and quinolone resistance-determining region (QRDR) of the *gyr*A, *rps*L, and *cme*R genes, was determined using ResFinder (Center for Genomic Epidemiology) with settings of a threshold of 85% identity and a minimum length of 60% [35].

### 2.4. Whole-Genome Sequencing

The extraction of genomic DNA was performed as above and the sequencing library was prepared with the Illumina TruSeq Nano DNA Kit, as per the manufacturer’s instructions with a library size of 350 bp. WGS was performed by Illumina HiSeq X technology at Macrogen (Seoul, South Korea) with a read length of 151 bp. The pair-ended reads passed the quality control check, followed by adapter trimming and quality filtering using Trimmomatic (v0.36) [36].

### 2.5. Construction of Phylogenetic Tree

The genome sequences (from our study) and those collected from public databases (Table 2 were uploaded to the Type (Strain) Genome Server (TYGS), a free bioinformatics platform available online: https://tygs.dsmz.de (accessed on 19 February 2021), for a whole genome-based taxonomic analysis [37]. TYGS employs the Genome-BLAST Distance Phylogeny method (GBDP) [38] to compare whole-genome sequences at the nucleotide level, allowing to calculate the digital DNA-DNA hybridization (dDDH) value and construct the phylogram. Submitted genomes were compared against all type strain genomes available in the TYGS database via the MASH algorithm, a fast approximation of intergenomic relatedness [39], and the 10 type strains with the smallest MASH distances were chosen per submitted genomes. An additional 10 closely related type strains selected by RNAmmer [40] were determined via the 16S rDNA gene sequences, and each sequence was subsequently BLASTed against the 16S rDNA gene sequences of type strains available in the TYGS database [41]. Intergenomic distances were used to infer a balanced minimum evolution tree with branch support via FASTME 2.1.4 including SPR post-processing [42]. Branch support was inferred from 100 pseudo-bootstrap replicates each. The trees were rooted at the midpoint [43] and visualized with PhyD3 [44]. The type-based species clustering using a 70% dDDH radius around each of the 13 type strains was done as previously described [37]. Subspecies clustering was done using a 79% dDDH threshold as previously introduced [45].

### 2.6. Data Analysis

The MIC values were interpreted using epidemiological cut-off values of the European Committee for Antimicrobial Susceptibility Testing (EUCAST, http://www.eucast.org, accessed on 28 November 2020).

BioEdit software (version 7.2.6.1) (http://www.mbio.ncsu.edu/BioEdit/bioedit.html, accessed on 19 February 2021) was used to edit, align, and analyze the DNA chromatograms [46]. A BLAST search was performed to compare consensus sequences (*gyr*A and *tet*(O)) with those from the GenBank database. Standard sensitive strains (L04566.1 and U63413.1) and resistant strains (KX982339.1 and MT176401.1) for *gyr*A were used for comparison. For the *gyr*A gene, the comparison was performed with Clustal Omega [47]. Amino acid sequences were deduced from the DNA sequences using the ExPASyTranslate tool [48].

For bioinformatics analysis, the filtered reads were mapped to reference genomes (NCTC11168 and NCTC11366) using Burrows-Wheeler Aligner (BWA-MEM), followed by variants identification and annotation. Produced mass sequence data were used to search for genetic variation based on the NCBI reference genome. After removing duplicates with Sambamba (v0.6.7) [49] and identifying variants with SAMTools [50], information on each variant was gathered and classified. SnpEff [51] was used to predict the variant effect at the protein level. Data was paired and assembled using SKESA assembler [52] while Quality Assessment Tool for Genome Assemblies QUAST [53] was used for assembly statistics and the genomes were annotated using Prokka [54]. Acquired AMR genes and point mutations conferring resistance to antimicrobials were searched using Abricate (https://github.com/tseemann/abricate, accessed on 22 January 2021) and NCBI’s AMRFinderPlus database [55]. Virulence genes were screened with VFDB [56]. The genomes deposited in GenBank were further annotated with PGAP version 5.1 [57]. GenBank accession numbers JAFETJ000000000 and JAFETK000000000 for *C. jejuni* and *C. coli*, respectively, were given after submission.

## 3. Results

### 3.1. Antimicrobial Resistance Profiles

The phenotypic AMR results revealed high-level resistance of *C. jejuni* strain 200605 and *C. coli* strain 200606 to ciprofloxacin (CIP), nalidixic acid (NAL), and tetracycline (TET) with MIC values ranging between 32 μg/mL and 128 μg/mL. Also, *C. jejuni* strain 200605 and *C. coli* strain 200606 were sensitive to erythromycin (MIC: 1 vs. 2 µg/mL), and gentamicin (MIC: 0.25 vs. 1 µg/mL), respectively.

PCR confirmed the presence of DNA of *gyr*A and *tet*(O) genes, but no band was seen for *cme*B. WGS confirmed the presence of the C257T point mutation in the quinolone resistance-determining region of the *gyr*A gene of both strains. Abricate and Resfinder [35] confirmed the phenotypic data related to FQ-resistance (C257T mutation). Furthermore, *tet*(O/32/O) and *tet*(O) genes associated with resistance to doxycycline, tetracycline, and minocycline were found in both isolates by the WGS. Apart from *bla*_OXA-452_ found in both isolates, *C. jejuni* strain 200605 also showed the bla_OXA-521_ and *bla*_OXA-193_ genes. The detection of PointFinder genes returned mutations in *gyr*A and 23S rRNA genes, but no mutations were found in *cme*R and *rps*L for *C. jejuni* strain 200605. Conversely, *cme*R was not detected, while *rps*L was found but without a mutation for *C. coli* strain 200606. The latter also showed 12 point mutations in 23S rRNA. Mass screening of contigs of both isolates using ABRicate also showed resistance to cephalosporin, penam, and the presence of *cme*B (efflux pump) conferring resistance to different antimicrobials.

### 3.2. Whole-Genome Sequencing Data

The annotation of the *C. jejuni* strain 200605 genome with PGAP returned 116 contigs: 1808 genes, of which 1688 were CDSs (with protein), 41 were RNAs (35 tRNAs, 3 ncRNAs, 1 rRNA), and 79 were pseudogenes (67 frame-shifted genes, 11 incompletes, 15 internal stops, and 13 multiple problems). 

The annotation of the *C. coli* strain 200606 genome returned 29 contigs: 1,865 genes, of which 1743 were CDSs (with protein), 42 were RNAs (36 tRNAs, 3 ncRNAs, and 1 rRNA), and 80 were pseudogenes (62 frame-shifted genes, 16 incompletes, 12 internal stops, and 7 multiple problems). Additional details of both strains are given in Table 3 and were made publicly available on BioProject PRJNA694501.

Of the called variants (Table 3), SNPs, insertions, deletions, transitions, and transversions were 21,816; 231; 219; 18,333; and 3483, and 45,561; 284; 257; 32,766; and 12,795 for *C. jejuni* strain 200605 and *C. coli* strain 200606, respectively.

### 3.3. Virulence Genes

*C. jejuni* strain 200605 and *C. coli* strain 200606 showed 87 and 57 virulence genes, respectively (Supplementary File S1). Adhesion factors (*cad*F, *peb*A, and *jlp*A), a cytolethal distending toxin (*cdt*ABC), invasion genes (*cia*B, *cia*C), and a biofilm formation gene (*ept*C) were only found in *C. jejuni* strain 200605 and not in *C. coli* strain 200606. However, both strains harbor genes coding for lipooligosaccharides (LOS), lipopolysaccharides (LPS), capsular (*gmh, waa*, and *kps* genes), chemotaxis (*che*A, *che*V, *che*W), and motility (*flh*, *fla*, *flg, ptm*) factors.

### 3.4. Phylogenetic Analysis

**Figure 1:**
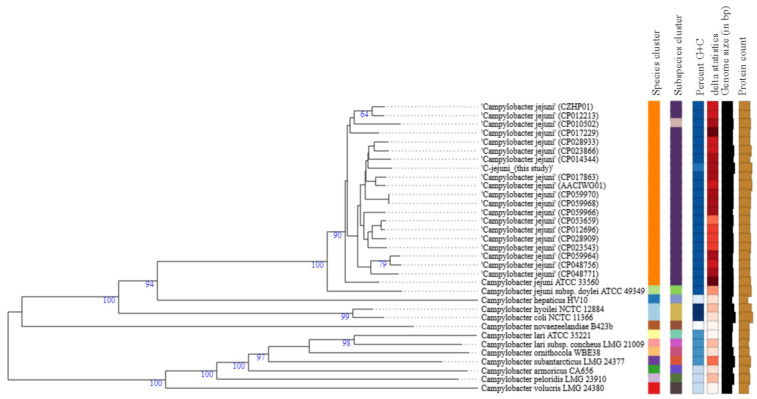
Type (Strain) Genome Server (TYGS) result for *C. jejuni* strain 200605 dataset. Tree inferred with FastME 2.1.4 [42] from GBDP distances calculated from genome sequences. Branch lengths are scaled in terms of GBDP distance formula d_5_; numbers above branches are GBDP pseudo-bootstrap support values from 100 replications. Percent G+C (27.39–30.98); δ statistics (0.138–0.286); protein content (1379–2041).

The Genome BLAST Distance Phylogeny (GBDP) approach used to generate a phylogenomic tree (Figure 1) shows that *C. jejuni* strain 200605 forms a cluster with CP014344, which was isolated from a human in South Africa. It is also closely related to other strains of chicken origin from several countries including South Korea (CP028933), the USA (CP023866, CP017863), and China (CP059968, CP059970). However, it is separated from another cluster of CP059964 (chicken) and CP048756 (duck), both from China (Figure 1). There were no differences among the species, subspecies, and percent G+C data of all *C. jejuni* strains used to generate the tree except for the *C. jejuni* (CP010502) strain that was isolated from human blood in Finland. The genome size was slightly higher compared to isolates from Type (Strain) Genome Server (TYGS), and it varied from 1.48–1.94 Mbp.

**Figure 2:**
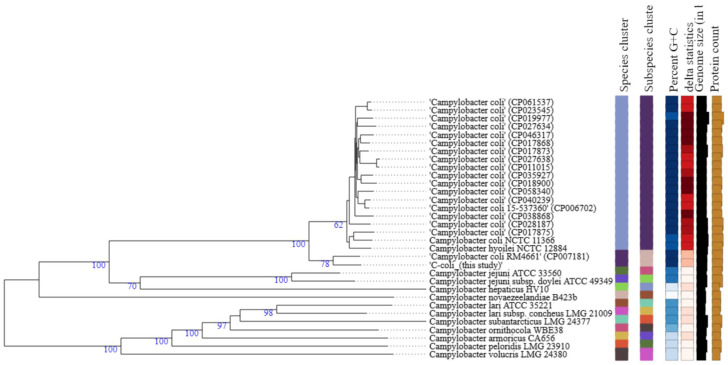
Type (Strain) Genome Server (TYGS) result for *C. coli* strain 200606 dataset. Tree was generated as for *C. jejuni*. Percent G+C (27.39–31.5); δ statistics (0.137–0.295); protein content (1379–2162).

The GBDP phylogenomic tree (Figure 2) shows that *C. coli* strain 200606 formed a separate cluster (species and subspecies) along with *C. coli* (CP007181) that was isolated from turkey and belongs to the same ST-1150 as the isolate of this study. Also, δ values were lower (0.181–0.175) than values for the cluster at the top of the tree (>0.2) (Figure 2). The overall treelikeness of the data set appeared to be high (low δ values). Briefly, δ statistics calculated using distance matrices allow for assessing the impact of individual operational taxonomic units (OTUs) on overall treelikeness (the lower the δ values, the better the treelikeness) [37].

### 3.5. Multilocus Sequence Typing (MLST)

*C. jejuni* strain 200605 belongs to ST-5229. So far, ST-5229 has not been assigned to a given clonal complex (CC). *C. coli* strain 200606 belongs to ST-5935, which belongs to CC-1150.

## 4. Discussion

Although the prevalence of *Campylobacter* spp. in table eggs is low, there is limited knowledge of their prevalence and ecology in layer chickens. Also, studies on the antimicrobial resistance profiles of layer chicken-derived *Campylobacter* isolates are limited [7,32]. This implies that the available data on whole-genome sequences of *Campylobacter* from layers important for epidemiological studies are also scanty.

This study highlights the genomic characterization and phylogenetic analysis of two FQ-resistant strains from layers in Gangneung. The isolates showed increased resistance to FQs. The resistance to ciprofloxacin has been attributed to two loci that were found in our isolates. The first one is the C257T point mutation in the *gyr*A gene, while the second factor is the *cme*ABC operon coding for an efflux pump [9,58]. Increased resistance of *Campylobacter* strains to FQs has been previously reported in Korea [59,60] and worldwide [9,61], but these strains are known to be highly persistent, even in the absence of the use of FQs [62,63]. The wide use of some FQs (enrofloxacin) in poultry farming has been associated with the spread of resistant *Campylobacter* strains and may explain the increasing resistance trend [60,64,65]. FQ-resistant *Campylobacter* strains have been classified by the World Health Organization (WHO) as high-priority antibiotic-resistant pathogens for which new drugs are required [66,67].

Ciprofloxacin and erythromycin have been used as the drugs of choice for treating *Campylobacter* infections [68]. The global distribution of ciprofloxacin-resistant strains has led to the adoption of erythromycin as the appropriate drug for campylobacteriosis therapy due to a limited number of macrolide-resistant strains [61]. Both strains of this study were sensitive to erythromycin and the WGS confirmed the results due to a lack of responsible point mutations (2074 and 2075) in the V domain of the 23S rRNA gene [69]. The reduced resistance to macrolides in *Campylobacter* strains from poultry may be associated with the limited use of macrolides in poultry production. Tylosin is used in swine or cattle, but not in poultry [70,71]. However, Sub-Saharan Africa (SSA) has recorded a lower prevalence of *Campylobacter* strains that are ciprofloxacin-resistant compared to erythromycin-resistant ones [68,72].

Phenotypic and genomic data showed resistance to tetracycline, which concurs with previous findings all over the world [59,73,74]. Higher resistance to tetracycline has been associated with the *tet*(O) gene coding for the ribosomal protection protein TetO [19] found in various Gram-positive and Gram-negative bacteria [63]. Moreover, tetracycline is overused in chicken and swine industries due to its affordability, and simple administration via drinking water [75]. It is worth noting that the chicken body temperature (42 °C) favors the conjugation process and thus contributes to the sharing of plasmids carrying various antimicrobial-resistant genes [76].

*Campylobacter* spp. are known to be inherently resistant to β-lactams including ampicillin [70], and we did not test for ampicillin resistance by broth microdilution. However, the WGS showed the presence of *bla*_OXA-452_, _521_, and _193_ genes which are inherent to *Campylobacter*. Ampicillin resistance is mainly due to enzymatic inactivation by *bla*_OXA-61,_ but other factors like porins and reduced affinity to penicillin-binding protein (PBP) have also been reported [70,77]. The isolates of the current study were sensitive to gentamicin, which corroborates previous reports [78,79,80]. However, higher resistance was reported in China for *C. coli* strains [74]. The limited resistance to gentamicin has been associated with its limited use to only systemic infections [81,82] and it is not used in poultry production [79]. Both ABRicate and ResFinder did not yield any resistance to streptomycin, as the *rps*L was found but without mutation. Surveillance of gentamicin-resistant strains should be performed in response to the increasing number of resistant strains as reported in the USA and China [61].

This study revealed that adhesion (*cad*F, *peb*A, and *jlp*A), invasion (*cia*BC), toxin (*cdt*ABC), *flg*SR two-component system, and biofilm formation (*ept*C) factors were only found in the *C. jejuni* strain 200605 genome and not in the *C. coli* strain 200606 genome. These factors highlight the virulent nature of the *C. jejuni* strain compared to *C. coli* which concurs with the literature [83]. Both strains expressed various other virulence factors involved in pathogenesis, like chemotaxis (*che*A, V, W), LOS, LPS, and capsule formation (*gmh, waa*, and *kps* genes). The mentioned genes contribute to the pathogenicity of *Campylobacter* strains while infecting humans, as they are all required for successful colonization and survival [15,84] of the bacteria within the host. Studies demonstrated that mutant *Campylobacter* strains were negatively affected in absence of some important genes [13,66]. For instance, *Campylobacter* strains lacking *cdt*B and *cdt*C were not cytotoxic, had reduced colonization, and had extra-intestinal invasiveness [15,85]. Flagellar genes (*fla*A, *fla*B, *flg*B, *flg*E, and *fla*C) are involved in various cell functions, like motility and biofilm formation [86,87]. The presence of capsular genes (*kps* D, E, F, C, S, T) and LPS associated gene (*hld*E) in both strains underline their virulence potential. The role of the capsule in the pathogenesis of *Campylobacter* has not been well defined, but it is suspected to interact with the mucus layer during adhesion, and it helps with intracellular survival [12]. *Hld*E is involved in protein glycosylation and correct LPS configuration [88]. Surprisingly, the *C. coli* strain 200606 harbored additional genes (*cj*1420c; *cj*1419c, *cj*1417c, *cj*1416c) involved in capsule biosynthesis [89] for *C. jejuni,* suggesting an exchange of some genes between *C. jejuni* and *C. coli* species. However, the introgression of *C. coli* by *C. jejuni* is not new [90]. Taken together, WGS data highlights the virulence profiles of study strains, which may give a clue to their respective pathogenicity.

The GBDP phylogenomic tree showed that *C. jejuni* strain 200605 clustered together with another isolate previously found in chicken meat in Korea (CP028933), but it was distantly related to another strain of human origin also reported in Korea (CP017229). This suggests some host preference and adaptation in *Campylobacter*. A study in Japan highlighted a distant relationship between *C. jejuni* from wild crows and poultry, showing the possibility of divergence due to host adaptation [91]. On the contrary, *C. jejuni* strain 200605 clustered with CP014344 collected from humans in South Africa, which could not be justified by the current study. We speculate that travel may be a predisposing factor in the occurrence of such a phenomenon. However, the phylogenetic tree (Figure 1) shows that other factors like the species, subspecies, percent G+C, and δ statistics were comparable for most of the *C. jejuni* strains used to build the tree. *C. coli* strain 200606 clustered with *C. coli* (CP007181) isolated from turkey, and this cluster was distantly related to other *C. coli* strains used to build the tree. Both chickens and turkeys are domestic poultry, and it seems common to find both strains clustering together. Also, introgression of CP007181 by *C. jejuni* would explain the clustering together with *C. coli* strain 200606 of this study in which some *C. jejuni* genes were found. Furthermore, the analysis showed that other factors like the species, subspecies, percent G+C, and δ statistics were different from the values of other *C. coli* strains used to build the tree (Figure 2). *Campylobacter* is evolving at high speed due to many recombination events that could lead to specific niche adaptation and thus justifying the obtained diversity [71]. Differential responses to environmental factors and/or management practices have also been suggested to contribute to strain distribution among various niches [92].

*C. jejuni* strain 200605 belongs to ST-5229 which so far has not been assigned to any clonal complex. This ST may be specific to the region, as other isolates (n = 4) of the same ST have been previously collected from chickens in Korea [60], while one isolate was isolated from swine in China, as shown by the pubMLST website. There is a shortage of information on this ST and why it has not been reported in other parts of the globe. *C. coli* strain 200606 belongs to ST-5935, which is part of the CC-1150. This ST is not common, but it has been reported in *C. coli* of chicken origin in Vietnam [93]. The CC-1150 has also been reported as the predominant clonal complex among *C. coli* from chickens in China [94]. Further studies are needed to understand the particularities of both STs and why they are not widely distributed. We also recommend studies on the roles played by indoor and cage-free laying hens along with their environment in disseminating *Campylobacter* species to the environment.

A limitation in our study was a low number of sequenced strains due to limited resources. However, the phylogenetic trees included *Campylobacter* strains from various hosts and countries to indicate the taxonomic features of isolates used in this study.

## 5. Conclusions

The current study describes the WGS of *C. jejuni* strain 200605 and *C. coli* strain 200606 from layer chickens in Korea. Both strains showed C257T point mutation in *gyr*A and *cme*ABC operon often associated with quinolone resistance. The two strains also carry *tet*(O) genes associated with tetracycline resistance. The presence of various virulence factors involved in motility, adhesion, invasion, toxin production, and chemotaxis shows the pathogenic potential of the studied strains. Phylogenomics revealed that the two strains resemble other strains of poultry and human origins. *C. jejuni* strain 200605 and *C. coli* strain 200606 belong to less common STs and this warrants further investigation. To the best of our knowledge, this is the first report of WGS data from *Campylobacter* species from layer chickens in Korea. Special attention should be paid to FQ-resistant strains due to a limited number of available alternative treatments.

## Figures and Tables

**Table 1:** Primers used for species and antimicrobial resistance confirmation.

Target Gene	Direction	Sequence (5′-3′)	Amplicon Size	Annealing Temperature (°C)	Reference
16S rRNA	Forward	GGATGACACTTTTCGGAGC	816	55	[8]
	Reverse	CATTGTAGCACGTGTGTC			
*cj* *0414*	Forward	CAAATAAAGTTAGAGGTAGAATGT	161		
	Reverse	CCATAAGCACTAGCTAGCTGAT			
*ask*	Forward	GGTATGATTTCTACAAAGCGAG	502		
	Reverse	ATAAAAGACTATCGTCGCGTG			
*tet*(O)	Forward	GCGTTTTGTTTATGTGCG	559	55	[8]
	Reverse	ATGGACAACCCGACAGAAG			
*cjgyr*A	Forward	GCCTGACGCAAGAGATGGTTTA	454		
	Reverse	TATGAGGCGGGATGTTTGTCG			
*cme*B	Forward	TCCTAGCAGCACAATATG	241		
	Reverse	AGCTTCGATAGCTGCATC			

**Table 2:** Genomic features of strains submitted to the TYGS Database.

No	Strain number	Country/Region	Sample Type	Host	Isolation Source	Disease Association
1	*C. jejuni* (CP059968)	China/Henan	Mixed culture	Chicken	Cloacal swab	NA
2	*C. jejuni* (CP012696)	USA/Albany CA	NA	Chicken	Chicken breast from retail	NA
3	*C. jejuni* (CP048756)	China/Zhejiang	Cell culture	Duck	Meat	NA
4	*C. jejuni* (AACIWG01)	USA:TX	NA	Chicken	Feces	NA
5	*C. jejuni* (CP012213)	Finland	NA	Human	Feces	Invasive
6	*C. jejuni* (CP023866)	USA:VA	NA	Chicken	Carcass	NA
7	*C. jejuni* (CP028909)	United Kingdom: London	Mono isolate	Chicken	NA	NA
8	*C. jejuni* (CP023543)	USA:CA	NA	Chicken	Chicken breast	Missing
9	*C. jejuni* (CP017863)	USA: Tulsa	NA	Chicken	Liver	NA
10	*C. jejuni* (CP014344)	South Africa: Cape Town	NA	Human	NA	Enteritis
11	*C. jejuni* (CP053659)	Italy:Lozzo Atesino	Mono isolate	Chicken	Feces	NA
12	*C. jejuni* (CP028933)	South Korea	NA	Chicken	Meat	NA
13	*C. jejuni* (CP059966)	China/Henan	Mixed culture	Chicken	Cloacal swab	NA
14	*C. jejuni* (CP048771)	China/Zhejiang	Cell culture	Duck	Meat	NA
15	*C. jejuni* (CZHP01)	Spain/Madrid	NA	Chicken	Meat	NA
16	*C. jejuni* (CP059970)	China/Henan	Mixed culture	Chicken	Cloacal swab	NA
17	*C. jejuni* (CP059964)	China/Henan	Mixed culture	Chicken	Cloacal swab	NA
18	*C. jejuni* (CP010502)	Finland	Multi-isolate	Human	Blood	Yes
19	*C. jejuni* (CP017229)	South Korea: Seoul	NA	Human	Stool	Food poisoning
	***C. coli***					
1	*C. coli*(CP061537)	USA: Pennsylvania	NA	Chicken	NA	NA
2	*C. coli*(CP023545)	USA:CA	NA	Chicken	Chicken breast	NA
3	*C. coli*(CP019977)	United Kingdom: Lincolnshire		Organic chicken farm		
4	*C. coli*(CP027634)	Germany: Berlin	NA	Turkey	Meat	Colonization
5	*C. coli*(CP046317)	USA: VA	NA	Human	Gastrointestinal tract	Unknown
6	*C. coli*(CP017868)	USA: Tulsa	NA	Chicken	Chicken liver from retail	NA
7	*C. coli*(CP017873)	USA: Tulsa	NA	Chicken	Chicken liver from retail	NA
8	*C. coli*(CP027638)	Germany: Berlin	NA	Turkey	Meat	Colonization
9	*C. coli*(CP011015)	United Kingdom: Cambridge	NA	Human	Feces	NA
10	*C. coli*(CP035927)	USA	NA	Chicken	Carcass	NA
11	*C. coli*(CP018900)	USA: Wyndmoor, Pennsylvania	NA	Chicken	Carcass/Retail	NA
12	*C. coli*(CP058340)	USA	Cell culture	NA	Environmental	NA
13	*C. coli*(CP040239)	United Kingdom: Sutton Bonington	NA	Cattle	Slurry	NA
14	*C. coli*(CP006702)	United Kingdom	Monoisolate	Human	NA	Gastroenteritis
15	*C. coli*(CP038868)	China: Shanghai	NA	Chicken	Cecum	NA
16	*C. coli*(CP028187)	Denmark	NA	NA	Missing	NA
17	*C. coli*(CP017875)	USA: Tulsa	NA	Pig	Pork	NA
18	*C. coli*(CP007181)	Missing	NA	Turkey	Missing	Missing

NA: not applicable.

**Table 3:** Genome characteristics and accession numbers of *C. jejuni* and *C. coli* strains.

Strain	SRA Accession No.	Reference Length	Mapped Site	Total Read	Mapped Read	Variant	GC (%)	Q30 (%)
*C. jejuni* strain 200605	SAMN17525986	1,641,464	1,596,540	9,800,132	8,152,436	22,266	30.12	97.48
*C. coli* strain 200606	SAMN17525987	1,938,580	1,584,482	9,922,508	8,616,294	46,102	31.19	97.27

## Data Availability

Datasets generated and/or analysed during the current study are available in GenBank repository under the BioProject number PRJNA694501 (https://www.ncbi.nlm.nih.gov/bioproject/PRJNA694501/). C. jejuni strain 200605 sequence accession number is JAFETJ000000000 (https://www.ncbi.nlm.nih.gov/nuccore/JAFETJ000000000.1/) while *C. coli* strain 200606 accession number is JAFETK000000000 (https://www.ncbi.nlm.nih.gov/nuccore/JAFETK000000000.1/).

## Supplementary Materials

The following are available online at https://www.mdpi.com/article/10.3390/genes12081131/s1, Supplementary File S1: Virulence genes.
