# Supplementary material for: Genomic Characterization of Fluoroquinolone-Resistant Thermophilic Campylobacter Strains Isolated from Layer Chicken Feces in Gangneung, South Korea by Whole-Genome Sequencing
Source: Genes (Basel). 2021 Jul 25;12(8):1131. doi: 10.3390/genes12081131 (PMC8391547; doi:10.3390/genes12081131)
Supplement: Supplementary file 1 [file genes-12-01131-s001.zip › Supplementary Table S1_Virulence genes.pdf]

Supplementary Table S1: Virulence genes

A. Virulence genes of *C. jejuni* strain

No.	GENE	% COVERAGE	% IDENTITY	ACCESSION	ROLE
1	pebA	100	99.23	NP_282073	Adhesion
2	cadF	100	98.75	NP_282616	Adhesion
3	jlpA	100	98.39	NP_282133	adhesin
4	ciaB	100	99.13	NP_282066	Invasion
5	ciaC	100	98.75	YP_002344633	Invasion
6	flaC	100	99.87	NP_281892	Invasion
7	flaD	100	96.94	NP_282040	Flagella biosynthesis
8	flhA	100	99.86	NP_282036	Flagella biosynthesis, flagellar type III secretion system (T3SS)
9	fliP	100	99.18	NP_281981	Flagella biosynthesis, flagellar type III secretion system (T3SS)
10	fliR	100	98.83	NP_282326	Flagella biosynthesis, flagellar type III secretion system (T3SS)
11	flgS	100	99.71	YP_002344200	flgSR two-component system (TCS) for flagellar gene expression
12	flgR	100	99	NP_282174	flgSR two-component system (TCS) flagellar gene expression
13	flgA	100	99.7	YP_002344176	Flagella biosynthesis, motility, and involved in biofilm formation
14	flaA	99.77	92.91	NP_282485	Flagellin gene
15	flaB	99.77	93.6	NP_282484	Flagellin gene
16	flgG	100	100	NP_281870	Flagellar basal-body rod protein
17	flgF	100	99.63	NP_281869	Length control of polar flagella (negatively regulating FlaA filament assembly)
18	flgH	100	99.71	NP_281859	Flagellar L-ring protein
19	pseE/maf5	100	97.08	NP_282483	Flagellin glycosylation/motility accessory factor
20	fliN	100	99.35	NP_281542	Flagellar motor switch protein,C-ring
21	fliG	100	99.32	NP_281510	C-ring
22	motA	100	98.71	NP_281528	flagellar rotation
23	motB	100	99.73	NP_281527	flagellar rotation
24	flhB	100	99.91	NP_281526	flagellar export apparatus (secretion)
25	fliH	100	99.76	NP_281511	flagellar export apparatus (secretion)
26	fliF	100	99.05	NP_281509	Rotor
27	cheV	100	99.79	YP_002343726	Chemotaxis protein
28	cheA	100	99.26	YP_002343725	Chemotaxis protein
29	cheW	100	100	YP_002343724	Chemotaxis protein

30	cheY	100	99.75	YP_002344511	chemotaxis protein
31	pseB	100	97.11	NP_282439	Pse biosynthetic pathway
32	pseC	100	95.4	NP_282440	Pse biosynthetic pathway
33	pseF	100	95.42	NP_282457	Pse biosynthetic pathway
34	pseG	100	98.91	NP_282458	Pse biosynthetic pathway
35	pseH	100	94.09	NP_282459	Pse biosynthetic pathway
36	pseA	100	99.3	NP_282462	Pse biosynthetic pathway
37	pseI	99.32	95.61	NP_282463	Pse biosynthetic pathway
38	ptmB	100	99.86	NP_282477	flagellin proteins post translational modification
39	ptmA	99.74	97.79	NP_282478	flagellin proteins post translational modification
40	pflA	100	99.79	NP_282693	component of medial disk
41	fliQ	100	98.52	NP_282802	flagellar export apparatus
42	flgK	100	99.18	NP_282606	Flagellar hook-associated protein
43	fliD	99.07	97.2	NP_281732	Flagellar hook-associated protein 2
44	flgM	100	98.99	YP_002344846	Anti-σ-factor
45	flgJ	100	99.12	YP_002344845	Rod assembly protein
46	flgI	100	99.05	NP_282602	Flagellar P-ring protein
47	kpsM	100	90.93	NP_282588	Capsule polysaccharide ABC transporter permease
48	kpsT	100	93.97	NP_282587	Capsule polysaccharide ABC transporter ATP-binding protein
49	kpsE	100	97.41	NP_282586	Capsule polysaccharide ABC transporter permease
50	kpsD	100	98.73	NP_282585	Capsule polysaccharide export system periplasmic protein
51	kpsF	99.58	94.07	NP_282584	CPS biosynthesis
52	hddA	100	94.51	NP_282566	putative D-glycero-D-manno-heptose 7-phosphate kinase
53	gmhA2	100	94.55	NP_282565	phosphoheptose isomerase
54	gmhA	100	94.12	NP_282296	Phosphoheptose isomerase
55	gmhB	100	95.73	NP_282299	D-glycero-alpha-D-manno-heptose-1,7-bisphosphate 7-phosphatase
56	kpsC	95.31	95.09	NP_282555	Capsule polysaccharide modification protein
57	kpsS	100	98.56	NP_282554	CPS biosynthesis
58	fliL	100	97.95	NP_282549	Motor
59	flgQ	100	99.33	YP_002344420	Flagellar-associated protein FlgQ
60	flgP	100	98.26	YP_002344421	Flagellar biosynthetic protein FliP
61	fliW	100	100	YP_002344468	flagellar assembly protein

62	waaC	100	98.25	NP_282281	Heptosyltransferase I
63	htrB	100	97.52	NP_282282	acyltransferase
64	Cj1135	98.97	95.04	NP_282283	glucosyltransferase
65	waaV	95.27	91.73	NP_282294	Lipooligosaccharide biosynthesis glycosyltransferase
66	waaF	99.79	93.42	NP_282295	ADP-heptose--LPS heptosyltransferase II
67	hldE	100	93.72	NP_282297	phosphorylation of D-glycero-D-manno-heptose 7-phosphate; ADP transfer from ATP to D-glycero-beta-D-manno-heptose 1-phosphate
68	hldD	100	97.69	NP_282298	ADP-glyceromanno-heptose 6-epimerase
69	fliI	100	98.41	NP_281405	H(+)-transporting two-sector ATPase
70	eptC	100	97.92	YP_0023436 98	Lipid A/FlgG phosphoethanolamine transferase
71	rpoN	100	99.04	YP_0023440 93	RNA polymerase sigma-54 factor
72	fliS	100	100	NP_281733	Flagellar secretion chaperone
73	flaG	100	99.45	NP_281731	Flagellar protein
74	flgB	100	97.69	NP_281712	Flagellar basal body rod protein
75	flgC	100	99.39	NP_281711	Flagellar basal-body rod protein
76	fliE	100	99.33	NP_281710	Flagellar hook-basal body complex protein
77	fliK	99.83	99.67	NP_281263	Putative flagellar hook-length control protein
78	flgD	100	99.44	NP_281264	Basal-body rod modification protein
79	flgE	100	100	NP_281265	flagellar hook protein
80	fliY	100	99.88	NP_281274	Flagellar motor switch protein
81	fliM	100	98.43	NP_281275	Flagellar motor switch protein
82	fliA	100	99.86	NP_281276	RNA polymerase sigma factor for flagellar operon
83	flhG	100	99.77	NP_281278	Polar flagellar biogenesis
84	flhF	100	99.66	NP_281279	Flagellar biosynthesis protein
85	cdtC	100	100	NP_281290	toxin activity
86	cdtB	100	99.5	NP_281291	toxin activity
87	cdtA	100	99.5	NP_281292	toxin activity

B. Virulence genes of *C. coli* strain

No.	GENE	%COVERAGE	% IDENTITY	ACCESSION	ROLE
1	flaD	100	93.25	NP_282040	Flagella biosynthesis
2	flgC	100	91.11	NP_281711	Flagellar basal-body rod protein
3	flgB	100	90.97	NP_281712	Flagellar basal body rod protein
4	flaG	100	99.73	NP_281731	Flagellar protein
5	fliD	100	96.38	NP_281732	Flagellar hook-associated protein 2
6	fliS	100	91.47	NP_281733	Flagellar secretion chaperone
7	cheW	100	100	YP_002343724	Chemotaxis protein
8	cheA	100	93.77	YP_002343725	Chemotaxis protein
9	cheV	100	92.37	YP_002343726	Chemotaxis protein
10	fliN	100	95.15	NP_281542	Flagellar motor switch protein,C-ring
11	hddC	96.25	92.51	NP_282564	D-glycero-D-manno-heptose 1-phosphate guanosyltransferase
12	gmhA2	100	93.23	NP_282565	phosphoheptose isomerase
13	hddA	100	94.61	NP_282566	putative D-glycero-D-manno-heptose 7-phosphate kinase
14	kpsF	98.95	94.88	NP_282584	CPS biosynthesis
15	kpsD	100	99.28	NP_282585	Capsule polysaccharide export system periplasmic protein
16	kpsE	100	97.05	NP_282586	Capsule polysaccharide ABC transporter permease
17	kpsT	100	93.97	NP_282587	Capsule polysaccharide ABC transporter ATP-binding protein
18	flgJ	100	96.2	YP_002344845	Rod assembly protein
19	flgM	100	98.99	YP_002344846	Anti-σ-factor
20	flgK	100	94.31	NP_282606	Flagellar hook-associated protein
21	fliM	99.72	91.94	NP_281275	Flagellar motor switch protein
22	fliY	100	98.81	NP_281274	Flagellar motor switch protein
23	flgE	100	97.01	NP_281265	flagellar hook protein
24	flgD	99.32	98.98	NP_281264	Basal-body rod modification protein
25	fliK	49.36	98.2	NP_281263	Putative flagellar hook-length control protein
26	fliK	53.87	96.62	NP_281263	Putative flagellar hook-length control protein
27	pseB	100	94.43	NP_282439	Pse biosynthetic pathway
28	pseC	100	94.61	NP_282440	Pse biosynthetic pathway
29	pseF	100	95.71	NP_282457	Pse biosynthetic pathway
30	pseG	100	98.91	NP_282458	Pse biosynthetic pathway

31	pseH	100	93.88	NP_282459	Pse biosynthetic pathway
32	pseA	100	98.94	NP_282462	Pse biosynthetic pathway
33	pseI	100	97.77	NP_282463	Pse biosynthetic pathway
34	maf4	99.33	90.14	YP_0023447 24	Flagellin glycosylation
35	Cj1420c	100	99.87	NP_282561	capsule polysaccharide biosynthesis
36	Cj1419c	100	99.61	NP_282560	capsule polysaccharide biosynthesis
37	Cj1417c	100	98.67	NP_282558	capsule polysaccharide biosynthesis
38	Cj1416c	99.87	98.95	NP_282557	capsule polysaccharide biosynthesis
39	cysC	100	99.03	NP_282556	capsule polysaccharide biosynthesis
40	kpsC	99.95	95.32	NP_282555	Capsule polysaccharide modification protein
41	kpsS	100	98.65	NP_282554	CPS biosynthesis
42	fliL	100	99.63	NP_282549	Motor
43	flaA	100	93.56	NP_282485	Flagellin gene
44	flaB	100	94.49	NP_282484	Flagellin gene
45	pseE/ma f5	100	97.51	NP_282483	Flagellin glycosylation/motility accessory factor
46	ptmA	100	95.33	NP_282478	flagellin proteins post translational modification
47	ptmB	100	95.62	NP_282477	flagellin proteins post translational modification
48	waaV	96.85	93.37	NP_282294	Glycosyltransferase
49	waaF	98.02	94.93	NP_282295	ADP-heptose--LPS heptosyltransferase II
50	gmhA	100	95.19	NP_282296	Phosphoheptose isomerase
51	hldE	100	93.43	NP_282297	phosphorylation of D-glycero-D-manno-heptose 7-phosphate; ADP transfer from ATP to D-glycero-beta-D-manno-heptose 1-phosphate
52	hldD	100	98.01	NP_282298	ADP-glyceromanno-heptose 6-epimerase
53	flgH	100	98.71	NP_281859	Flagellar L-ring protein
